# Unlocking the Complexity of Mitochondrial DNA: A Key to Understanding Neurodegenerative Disease Caused by Injury

**DOI:** 10.3390/cells10123460

**Published:** 2021-12-08

**Authors:** Larry N. Singh, Shih-Han Kao, Douglas C. Wallace

**Affiliations:** 1 Center for Mitochondrial and Epigenomic Medicine, Division of Human Genetics, The Children’s Hospital of Philadelphia, Philadelphia, PA 19104, USA; wallaced1@chop.edu; 2 Resuscitation Science Center, The Children’s Hospital of Philadelphia, Philadelphia, PA 19104, USA; kaos@chop.edu; 3 Department of Pediatrics, Division of Human Genetics, Perelman School of Medicine, University of Pennsylvania, Philadelphia, PA 19104, USA

**Keywords:** mitochondria, traumatic brain injury, ischaemic stroke, evolution, genomics, genetics

## Abstract

Neurodegenerative disorders that are triggered by injury typically have variable and unpredictable outcomes due to the complex and multifactorial cascade of events following the injury and during recovery. Hence, several factors beyond the initial injury likely contribute to the disease progression and pathology, and among these are genetic factors. Genetics is a recognized factor in determining the outcome of common neurodegenerative diseases. The role of mitochondrial genetics and function in traditional neurodegenerative diseases, such as Alzheimer’s and Parkinson’s diseases, is well-established. Much less is known about mitochondrial genetics, however, regarding neurodegenerative diseases that result from injuries such as traumatic brain injury and ischaemic stroke. We discuss the potential role of mitochondrial DNA genetics in the progression and outcome of injury-related neurodegenerative diseases. We present a guide for understanding mitochondrial genetic variation, along with the nuances of quantifying mitochondrial DNA variation. Evidence supporting a role for mitochondrial DNA as a risk factor for neurodegenerative disease is also reviewed and examined. Further research into the impact of mitochondrial DNA on neurodegenerative disease resulting from injury will likely offer key insights into the genetic factors that determine the outcome of these diseases together with potential targets for treatment.

## 1. Introduction

Mitochondria are vital to eukaryotic cells, providing a supply of energy in the form of adenosine triphosphate (ATP) via the process of oxidative phosphorylation. The known roles of these organelles, however, involve an array of complex and diverse functions, including apoptosis, calcium signaling, reactive oxygen species signaling, and immune signaling [1,2]. It is no surprise then that mitochondrial dysfunction disrupts not only energy demanding processes, but several other cellular functions. Typically, mention of mitochondrial disease refers to primary mitochondrial disease, which entails defects in either mitochondrial or nuclear DNA that impair mitochondrial function or structure [3]. But mitochondrial dysfunction has also been implicated in several common human diseases including autism [4], glaucoma [5], and cancer [6]. Defects in mitochondrial function have also been hypothesized to play a role in the etiology of traditional neurodegenerative diseases, such as Alzheimer’s disease [7] and Parkinson’s disease [8].

Much less attention, however, has been devoted to examining the role of mitochondrial genetics in the progression of neurodegenerative diseases that are prompted by injury, such as traumatic brain injury (TBI) and ischaemic stroke. That mitochondrial genetics encompasses several key differences from Mendelian genetics further complicates the interpretation of results from mitochondrial genetic studies. To perform genetic disease associations in the context of mitochondria demands a deep understanding of the intricacies of mitochondrial biology along with the nuances of interrogating mitochondrial genetic variants. This review introduces and discusses these complexities to aid in understanding the influence of mitochondrial genetics on neurodegeneration due to injury.

## 2. Fundamentals of Human Mitochondrial Genetics

**Figure 1:**
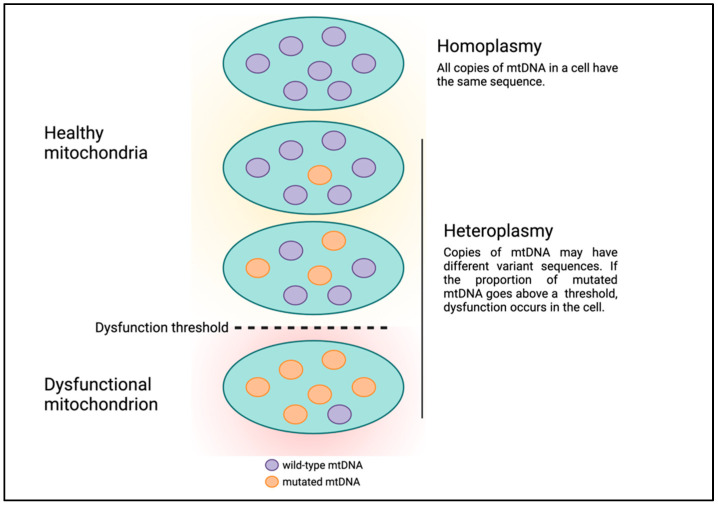
Heteroplasmy and the threshold effect.

Human mitochondrial DNA (mtDNA) is circular and comprises 16,569 bases which encode 13 protein-coding genes, in addition to 22 tRNAs and 2 rRNAs for translating these protein-coding genes [9]. Although mtDNA is much smaller than nuclear DNA (nDNA), there are several fundamental differences between Mendelian and mitochondrial genetics that can make understanding mitochondrial genetics abstruse. First, mtDNA is strictly maternally inherited and there are mechanisms in various organisms to degrade paternal mtDNA following fertilization [10]. Second, each human nucleated cell contains 100s to 1000s of mitochondria, and mtDNA is polyploid with each mitochondrion having multiple copies of mtDNA. For example, estimates of the number of mtDNA per mitochondrion in mouse cells ranged from one to more than ten, with a median of three [11]. Each copy of mtDNA can have unique, differing DNA sequences. The presence of multiple copies of mtDNA with different DNA sequences existing in the same cell or a population of cells and tissues is termed heteroplasmy (Figure 1) [12]. High and low levels of variant mtDNA sequences correspond to high and low heteroplasmy, respectively. Similarly, if all copies of mtDNA have the same sequence, this condition is referred to as homoplasmy, which corresponds to either 0 or 100% heteroplasmy. Presumably, mitochondria and cells can continue to function in the presence of low levels of mtDNA genetic defects. But as the level of heteroplasmy rises and genetic defects accumulate, the cell is unable to cope, leading to cell dysfunction or death, a phenomenon called the threshold effect (Figure 1) [13].

It is well known that animal eukaryotes possess two genomes, one nuclear and one mitochondrial. The question is, why have these two separate sources of DNA, and what role does mtDNA serve in the functioning of the cell? A follow-up question is how do genetic mutations in the mtDNA affect the cell’s function and health? To answer these questions and understand how variation in mtDNA contributes to disease pathology requires a deeper understanding of the origin of this unique and vital organelle. We also delve into the functional relevance of why mitochondria possess their own DNA separate from the nucleus.

## 3. Origins of Mitochondria and mtDNA

**Figure 2:**
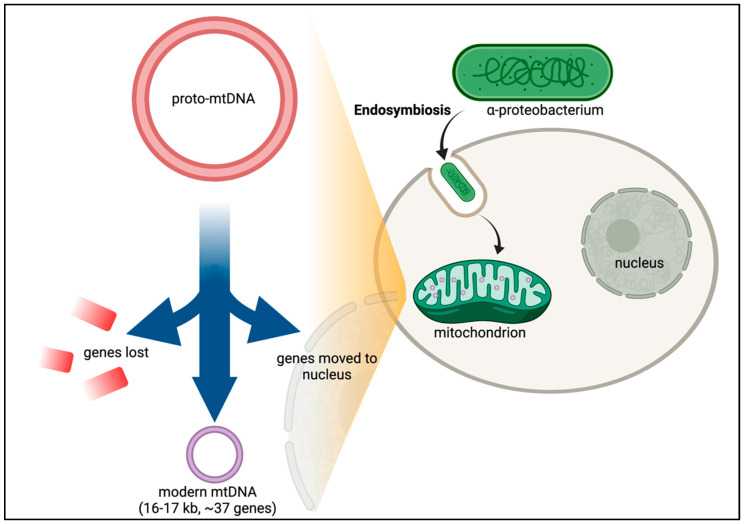
Endosymbiotic Theory and CoRR hypothesis. While the exact timing and emergency of eukaryotes is still unclear, recurrent gene transfer evidence supports the hypothesis that the original precursor to eukaryotic cells was a prokaryotic microbe that ingested an aerobic bacterium [20]. Following a period of evolution, the proto-mitochondrion would then have lost the genes which permitted adaptation of the free-living bacterium to environmental changes. Essential genes were also transferred from the bacterium into the nucleus. This gene transfer led to modern mtDNA in which only genes with core roles in the electron transport chain were retained in the mtDNA.

After many years of debate, it is now established that mitochondria in eukaryotic cells evolved via endosymbiosis from bacteria—most likely α-proteobacteria (Figure 2) [14,15,16]. Phylogenetic analysis of the mitochondrial proteome predicts that thousands of genes were lost from the ancestral α-proteobacteria in the transition to the proto-mitochondrion [17]. Of the estimated 800 human genes predicted to have evolved from α-proteobacteria, only about 200 are in the mitochondrial proteome, suggesting that the ancestral bacterial genes have adapted to perform other cellular functions [17]. Transferring the mitochondrial genes to the nuclear genome offers protection to mutations caused by the harsh mitochondrial environment, including the accumulation of deleterious mutations in an irreversible manner—Muller’s ratchet [18,19]. Other genes not of bacterial origin have also evolved to become part of the mitochondrial repertoire of genes. Over 99% of the proteins found in human mitochondria originate from genes encoded in nuclear DNA (nDNA). But despite the palpable advantages of transferring genes to the nucleus, the mitochondrion retains 13 protein-coding genes. Why would evolution retain this miniscule but essential set of genes along with a complete and independent translation machinery at such large expense?

Several hypotheses have been formulated to explain why mitochondria retain a small set of genes separate from the nuclear-encoded genes. The simplest of these hypotheses is the neutral theory of molecular evolution [21]. In the context of mitochondria, the neutral theory of molecular evolution postulates that gene retention and loss is random and does not affect the fitness of the mitochondria. This theory amounts to a null hypothesis of molecular evolution and fails to explain many of the salient features of mtDNA-encoded genes. Many other more elaborate hypotheses to explain the selective forces shaping the mitochondrial genome have been proposed [22]. One hypothesis with growing support is the “co-localization for redox regulation” or CoRR hypothesis [23]. The CoRR hypothesis proposes that the redox state of gene products within an organelle regulates the expressions of their corresponding genes.

To fully appreciate how the CoRR hypothesis applies to mitochondria, consider the most prominent feature of the mitochondrion—the electron transport chain (ETC) [24]. The ETC is comprised of a series of protein complexes that transfer electrons through redox reactions. Although the movement of these electrons is vital to the cell, certain by-products of the ETC, such as reactive oxygen species (ROS) are toxic [25]. To safeguard the cell from ROS and other harmful molecules, the electron carriers are compartmentalized within the mitochondria away from the rest of the cell. According to the CoRR hypothesis, the genes that are regulated by the redox state of these mitochondrial electron carriers must also be in the mitochondria. Accordingly, mitochondrial electron carriers include three respiratory chain complexes—complex I, III, and IV—all of which comprise proteins encoded in the mtDNA. But why must these particular genes be encoded in the mtDNA? Why are these genes not encoded in the nDNA instead?

Considering that, in most human cells, there are 100s to 1000s of mitochondria, having multiple mitochondria within a cell allows for both rapid response to energy demands and precision sensing of the micro-environment surrounding the mitochondrion. It also means that each mitochondrion has individual needs, requirements, and responses. The CoRR hypothesis suggests that the specific subset of genes encoded in the mtDNA must be precisely regulated by each individual mitochondrion. Alternatively, if these genes were encoded in the nDNA, then gene expression regulation would affect and respond to all mitochondria within the cell, which could lead to detrimental consequences. The redox balance within a mitochondrion is tightly regulated, and over- or under-expression of these genes would lead to mitochondrial and ultimately cell death.

The CoRR hypothesis explains why genes were retained in the mtDNA, but why would evolution select for these specific genes? A data-driven, phylogenetic analysis of protein-coding mtDNA-encoded genes from multiple species has revealed several important insights and answers to these questions [18]. First, genes important for the core assembly of mitochondrial ETC protein complexes tend to be retained in the mtDNA. This finding is consistent with the CoRR hypothesis and further suggests that the mitochondrion can respond to redox changes by creating a core ETC complex structure, and the peripheral subunits can be assembled to the core from nDNA-encoded proteins. Furthermore, this arrangement allows for localized regulation and control of the ETC complexes within each mitochondrion. Second, the study showed that the genes retained in mtDNA had high GC content and high protein hydrophobicity. We next consider the different types of mtDNA variation and how changes in these vital genes arise.

## 4. Mitochondrial DNA Variation

Changes and mutations in DNA sequences are inevitable, and mtDNA is no exception. Given the harsh environment within the mitochondrion, lesions and damage often arise. The prevailing hypothesis is that most mtDNA mutations occur due to free radical damage, but this assertion is questionable. Studies have shown that most mtDNA mutations result from spontaneous replication errors [26]. It has also been proposed that mtDNA is susceptible to DNA damage due to the lack of histones. This assertion is also dubious as mtDNA-bound proteins may offer a similar level of protection to histones [27]. There are six major causes of mtDNA lesions: (1) alkylation of nucleotides, (2) hydrolytic deamination of nucleotides, (3) adduction by carcinogens which can cause crosslinking of DNA, (4) replication errors, (5) single and double-stranded breaks in DNA, and (6) oxidative damage to nucleotides likely caused by reactive oxygen species (ROS) generated in the mitochondrion [28]. Conceivably, damage to mtDNA exists in low amounts resulting in low heteroplasmic variants. The percentages of these heteroplasmic variants may eventually increase and become fixed, resulting in homoplasmic variants. Thus, mitochondrial genetics follows the principles of population genetics. MtDNA variants can be either somatic or inherited, and many inherited homoplasmic mtDNA variants are in high linkage disequilibrium and are inherited as a haplotype or haplogroup [6]. The emergence of haplogroups in human evolution is thought to have permitted ancient human migrations, along with climate and environmental adaptation [24]. Haplogroups are also associated with risk for common human diseases including diabetes, cardiovascular disease, and cancer [6,29].

## 5. Quantifying Mitochondrial DNA Variation

To determine which mtDNA variants are associated with human disease obviously requires accurate assessments of mtDNA variants. In clinical settings the standard approach is to perform mtDNA amplification by long-range PCR amplification followed by NGS and variant calling [30]. To further enrich mtDNA, samples can be treated with exonuclease V to digest linear DNA, leaving the circular mtDNA intact [31]. If enriched mtDNA sequence is unavailable, there are several approaches for performing retrospective assessment of mtDNA from existing genetic studies that have originally focused on nDNA. For example, DNA microarrays are typically designed to interrogate millions of nDNA variants, yet contain a subset (typically less than 200) of mtDNA single nucleotide variants (SNVs). These DNA microarrays may be used to identify both homoplasmic and heteroplamic mtDNA SNVs [32]. The resulting SNVs can also be used to compute haplogroups. Care must be taken, however, since not all haplogroups, and especially subhaplogroups, can be accurately computed from a subset of mtDNA SNVs. Naturally the more SNVs that are interrogated, the better the accuracy and resolution of haplogroups. Hence, one author (L.N.S.) has introduced an approach of computing haplogroups from known mtDNA sequences using only the subset of SNVs interrogated, to assess the accuracy and ability of these SNVs to identify haplogroups [4].

Today, genomic data typically constitutes next-generation sequencing (NGS) data, and in particular whole exome sequencing and whole genome sequencing (WES and WGS, respectively). WES entails capturing RNA-coding regions of the genome using DNA or RNA baits that hybridize to the DNA of interest. The resulting captured or enriched regions are then sequenced using high-throughput NGS techniques. Due to the abundance of mtDNA in human cells, WES also yields a substantial amount of mtDNA sequence. Off-target DNA enrichment including mtDNA, however, is not random and varies depending on the capture kit used and sequence content [33]. In particular, low-level heteroplasmy may appear as homoplasmy in regions of the mtDNA that have enrichment biases. Consequently, custom WES assays have been designed with probes specific to the mtDNA to account for these problems [34]. These custom arrays capture a subset of nDNA-encoded coding genes and mtDNA sequence.

**Figure 3:**
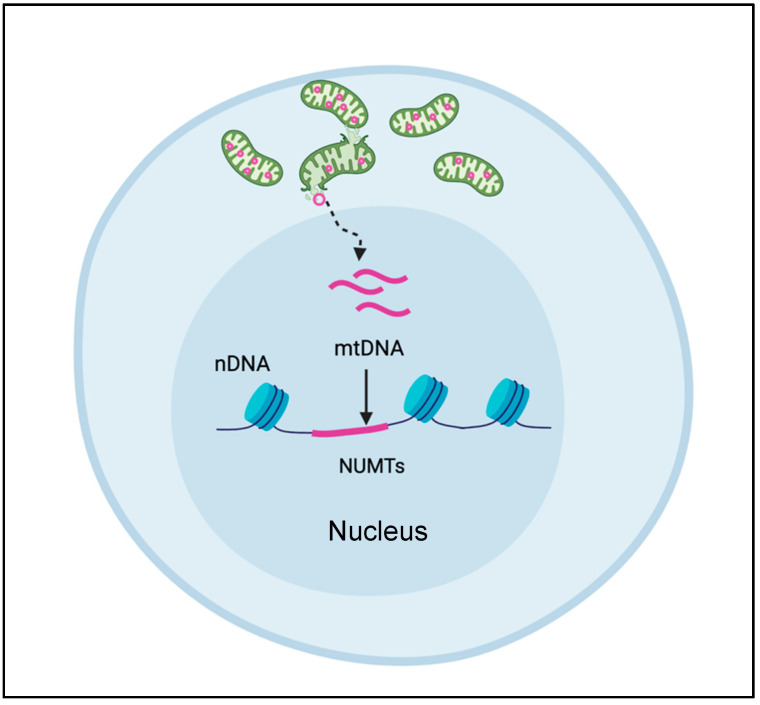
NUMT formation. Nuclear encoded mitochondrial sequences (NUMT) arise when mtDNA sequences are released from the mitochondrion and enter the nucleus to become incorporated into the nuclear DNA.

Compared to WES, WGS is less prone to enrichment biases. But the success of WGS, like all NGS methods, relies heavily on the ability to align short DNA sequence reads accurately and uniquely. The uniqueness of these alignments is complicated by the presence of nuclear encoded mitochondrial DNA sequences (NUMTs, pronounced “new mights”) that are homologous to mtDNA sequences (Figure 3). The transfer of mtDNA sequences into the nuclear genome is an ongoing, evolutionarily conserved process, producing various permutations of mtDNA, ranging from stretches of uninterrupted mtDNA sequences to re-arranged mtDNA segments originating from different individual mitochondria. The mechanism of how NUMTs arise or numtogenesis [35] is still up for debate and discussed extensively elsewhere. Occurring at a rate of about 17 bp per 100 kb of nuclear genomic sequence, for a total of over 400 kb of DNA sequence, NUMTs are not rare occurrences [35]. Moreover, the human genome contains over 700 inherited NUMTs ranging from 63 to 100% sequence identity [36], and none of the mtDNA sequence is immune to NUMT transfer [37,38].

**Figure 4:**
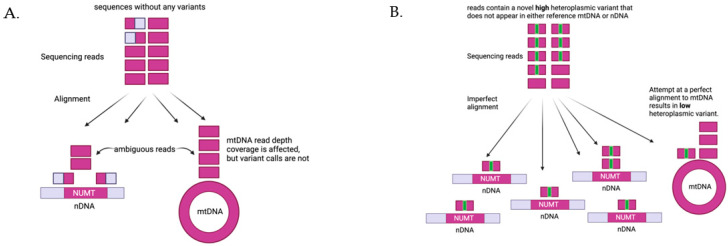
Impact of NUMTs on heteroplasmy quantification. (**A**) If there are no variants in the sequencing reads from mtDNA, the reads will align perfectly to either NUMTs in the nDNA or mtDNA. Reads shorter than the NUMTs sequences will align ambiguously to both NUMTs and mtDNA, and the alignment software has no way of determining exactly where the reads should go. There are several practical options to handle this case (1) drop non-unique alignments, (2) choose an alignment randomly to either NUMTs or mtDNA, or (3) favor alignments to mtDNA. None of these options will affect heteroplasmy computation--since there are no variants by definition, there cannot be any heteroplasmic variants. These options, however, will each give different and erroneous calculations for the read depth of mtDNA coverage. (**B**) Consider reads that originate from mtDNA and possess a novel variant (a variant that does not appear in either the reference nDNA or reference mtDNA). Alignments to either NUMTs or mtDNA will be imperfect, i.e., there will be mismatches. Furthermore, the alignment software cannot accurately determine whether the reads are from mtDNA or nDNA and will assign these reads to both. Again, there are several options, all of which are error prone. If the reads are aligned randomly or with preference to mtDNA, as in this figure, then the variant heteroplasmy level will be underestimated, as some reads will inaccurately be aligned to NUMT regions. The more copies of this NUMT there are, the more reads will erroneously align to NUMTs, thus further reducing the heteroplasmy estimate. Similar issues arise if the reads originally originated from NUMTs instead of mtDNA, as reads could erroneously align to the mtDNA, inflating the estimate of heteroplasmy.

To contend with the issues of computing mtDNA variants from WGS data, several computational methods have been developed with varying success [39,40]. NUMTs confound the computation of mtDNA variants, as it is not possible for alignment software to distinguish a NUMT from authentic mtDNA if the sequencing reads are shorter than the NUMT. Deeper sequencing does not alleviate this problem since there are systematic biases in the mtDNA sequences that alter coverage [41]. The computation of mtDNA heteroplasmy is further complicated and distorted if there are several copies of a NUMT, as each copy of a NUMT is a potential erroneous target for mtDNA alignment (Figure 4). Given the large number NUMTs in the human genome, having multiple copies of a single NUMT is not uncommon. We have recently developed MitoScape, a big-data, machine-learning workflow for accurately obtaining mitochondrial DNA from next-generation sequencing to contend with the issues of heteroplasmy and NUMTs [42].

Recently, single cell assay for transposase-accessible chromatin sequencing (scATAC-seq) has been modified to enrich mtDNA sequences for identifying mtDNA variants [43]. The rationale is that mtDNA has no chromatin and can be interrogated by scATAC-seq. Nevertheless, there are several concerns with this method. First, NUMT insertion in the nDNA is not random and favors open-chromatin regions [44], therefore NUMT regions are also likely to be enriched in scATAC-seq (Figure 3). Second, there is evidence of several proteins binding to the mtDNA, including TFAM which would potentially lead to non-uniform sequencing of mtDNA [45], leading to biases. Third, the short reads used in most single-cell sequencing techniques—10x Chromium—are likely too short to distinguish mtDNA from NUMTs, especially given the large number of NUMTs. There has been some attempt to reduce the impact of NUMTs on mtDNA variant analysis by filtering regions identified in databases of known NUMTs. This approach is flawed, however, since NUMTs are dynamic, vary from person to person, and can enter the nucleus during a person’s lifetime particularly under stress conditions; hence a static list of NUMTs is likely to lead to biases [46].

## 6. Mitochondrial DNA Copy Number

Genetic variation in mtDNA appears in similar forms as does that of nDNA, including single nucleotide variants (SNVs), and insertions and deletions (indels). All these variant types can exist in either heteroplasmic or homoplasmic states. Another form of variation that is unique to mitochondria is mtDNA copy number—the number of copies of mtDNA per cell. The exact biological function and interpretation of mtDNA copy number (mtDNA-CN), however, is unclear. Evidence suggests that mtDNA-CN is independent of either mitochondrial content or biogenesis, but may be a marker of mitochondrial dysfunction depending on the biological context [47]. MtDNA-CN has also been hypothesized to be a genotoxic stress signal by triggering nDNA damage and repair responses when mtDNA is released into the cytoplasm [48].

Several studies have reported an association between mtDNA-CN and human disease [26,49,50]. Direct evidence of an mtDNA-CN decrease resulting in a disease comes from mtDNA-depletion syndromes, but evidence in other pathologies is lacking [26]. For example, recent epidemiological studies suggest that mtDNA-CN changes are correlated with environmental influences in fetal life [49]. In bipolar disorder there have been mixed findings, with certain studies finding either a positive, negative, or no correlation between mtDNA-CN and bipolar disorder [50]. Similar observations have been reported in ageing and neurodegenerative disorders with no consistency in the direction of the correlation between mtDNA-CN and these disorders [26]. There are several factors altering mtDNA-CN which would potentially explain the discrepancies in findings. MtDNA-CN is affected by factors such as age, gender, mtDNA extraction method, cell type composition, and sample heterogeneity [26]. Cell type composition reflects the fact that the cell composition of tissue can be affected by factors such as age or pathology, and mtDNA-CN varies by cell type. For example, tumor cells likely contain a larger variety of cells compared to nearby healthy control cells within the same affected organ [26]. MtDNA-CN also varies by both tissue and developmental stage as mtDNA-CN is regulated in a tissue-specific manner and strictly controlled during development [51]. Sample heterogeneity arises due to different cell type composition of different samples of an investigated tissue. For example, mtDNA-CN is commonly measured from blood samples. Blood platelets, however, contain a large amount of mtDNA, and the inclusion of platelets can increase mtDNA-CN significantly. In short, to obtain accurate estimates of mtDNA-CN requires accounting for both white blood cell and platelet count, and reducing or eliminating platelets is preferred [52]. But the white blood cell composition matters, and specifically the relative ratios of granulocytes, monocytes, and lymphocytes, as each contains different values of mtDNA-CN [47,53].

## 7. Mitochondrial RNA

Finally, we visit a well-studied form of genetic regulation: gene expression and specifically, mitochondrial RNA (mtRNA). Homoplasmic mtDNA variants can exhibit incomplete penetrance in several mitochondrial disorders including Leber’s hereditary optic neuropathy (LHON) and sensorineural hearing loss (SNHL) [54]. Mitochondrial transcriptomics offers another layer of regulation between mtDNA and the resulting proteins. Furthermore, the mitochondrial transcriptome not only encodes messenger RNAs, but also the ribosomal and transfer RNAs required to translate these messenger RNAs. Changes in the expression levels of each type of gene will alter mitochondrial protein dynamics. Hence, mitochondrial RNA (mtRNA) expression may offer insight into the consequences of mtDNA variants and explain their variable presentations. MtRNA processing is a complex sequence of events as the mtDNA is transcribed as long polycistronic sequences and then spliced into shorter functional transcripts [55].

RNA editing serves as yet another layer of regulation, involving post-transcriptional changes to RNA sequences which can increase the repertoire of protein products in an organism. One form of RNA-editing in mammals, adenosine (A) to inosine (I) deamination, is catalyzed by the ADAR (adenosine deaminase acting on RNA) enzyme family [56]. RNA editing has been reported in human neurological diseases. For example, a decrease in A-to-I RNA-editing levels in brain tissues from patients with Alzheimer’s disease has been observed [57]. This decrease in editing was most apparent in the hippocampus and, to a lesser extent, in the temporal and frontal lobes. Mutations in RNA modification enzymes have been associated with several human diseases including cancer, cardiovascular disease, and mitochondrial disease (See [58] for an extensive discussion of RNA post-transcriptional modifications in human disease). RNA editing is both widespread and highly diverse in mitochondria [59]. The most common types of RNA editing in mitochondria are pyrimidine exchanges: cytidine (C) to uridine (U) and vice-versa, as well as A-to-I deamination. Although RNA editing has been reported in human mitochondria, it is unclear if any of these edits are pathogenic [60,61]. We hypothesize that mitochondrial RNA editing may also help explain the incomplete penetrance of homoplasmic disease-causing variants.

Yet another mode of regulation due to mtRNA is the presence of long non-coding RNAs (lncRNAs). LncRNAs are RNA molecules greater than 200 bp long that are not translated into proteins. The presence of mitochondrial-encoded lncRNAs (mt-lncRNAs) is controversial due to the difficulty of accurately distinguishing mt-lncRNAs from unprocessed mtRNA transcripts using short-read NGS. Nevertheless, the existence of three mt-lncRNAs (lncND5, lncND6, and lncCytb) in particular has been validated in several studies [62]. The function of these mt-lncRNAs is believed to be the regulation of mtRNA expression.

While the gene expression levels of mtDNA-encoded genes likely offer invaluable insight into the function and activity of the mitochondrion, quantifying mtRNA expression is non-trivial and differs significantly from quantifying nuclear RNA. First, not all mtRNA is polyadenylated and the length of a poly-A tail varies by cell type [63]. Hence, RNA capture by oligo-dT capture is not appropriate for mtRNA expression analysis. Total RNA or ribo-depletion is the preferred method, but ribo-depletion will render mitochondrial ribosomal RNA measurement inaccurate. Second, each strand of the mtDNA is transcribed, and there are overlapping genes on each strand, making strand-specific RNA sequencing a must. Third, mtRNA is transcribed as long polycistronic RNA molecules which are then processed to produce individual RNAs for each gene [64]. Thus, RNA within a mitochondrion comprises a mixture of processed and unprocessed transcripts and determining the relative proportions of each component is not trivial.

## 8. Secondary Neurodegenerative Disorders

Neurodegenerative diseases are disorders that progressively degrade central nervous system function and are often associated with ongoing neuronal loss, ultimately leading to cognitive or physical deficits [65,66]. Traditional neurodegenerative diseases include Alzheimer’s Disease and Parkinson’s Disease. But there is a group of neurodegenerative diseases which occur as a result of a primary insult or injury. These diseases are examples of secondary neurodegenerative diseases and include stroke and traumatic brain injury [67]. Traditionally, studies of the association between mtDNA variation and disease pathology have involved predicting the risk for acquiring a disease given that the person carries a particular mtDNA allele. Studying mtDNA-related secondary neurodegenerative disorders, particularly those resulting from injury, instead involves assessing the presence or absence of an mtDNA allele that affects post-injury disease progression and recovery. Two examples of such conditions are traumatic brain injury and ischaemic stroke; we examine the relationship between these disorders and human mtDNA variation.

### 8.1. Traumatic Brain Injury

Encephalopathies—disorders of the brain affecting brain function or structure—are conspicuous in primary mitochondrial disease. Two well-known mitochondrial diseases, mitochondrial encephalomyopathy, lactic acidosis, and stroke-like episodes (MELAS); and myoclonus epilepsy with ragged-red fibres (MERRF) are distinguished by severe disorders of the nervous system. These two diseases are also associated with point mutations in mtDNA: m.3243 > G/tRNA-Leu for MELAS, and m.8344A > G/tRNA-Lys for MERRF [68]. With regard to traditional neurodegenerative diseases, there is ample evidence linking mtDNA variation to disorders such as Alzheimer’s and Parkinson’s disease [69]. Less is known, however, about the involvement of mtDNA variation in neurodegeneration caused by injury. Traumatic brain injury (TBI) is often caused by a sudden blow to the head or body and is one type of acquired brain injury. There is a large amount of inter-patient variation in the outcome of TBI that cannot be adequately explained by the type or severity of injury and other obvious factors such as age and gender. Genetics may explain some of this variation, with the most well-studied gene in TBI being the nDNA-encoded Apolipoprotein (apo) E (APOE) gene [70]. APOE transports lipids and cholesterol in the brain, maintains synaptic integrity, promotes neural recovery and repair, and modulates inflammatory responses after central nervous system injury [71,72,73,74]. Three allelic variants of APOE in a single gene locus on human chromosome 19 yield three isoforms—APOE2, APOE3 and APOE4 [75]. Among these, APOE4 is the most neurotoxic isoform and induces neuropathology through many pathways, including mitochondrial pathways [75]. Furthermore, APOE4 can alter mitochondrial membrane potential [76], reduce mitochondrial motility [77], and decrease mitochondrial respiratory enzyme levels or activities [78].

Collectively, these findings suggest a role for mitochondria in the progression of age-related neurodegeneration. In terms of injury-related neurodegeneration, studies have demonstrated that TBI is associated with mitochondrial dysfunction in injured brain regions [79]. Following TBI, mitochondria potentially play a critical role in cell damage by various mechanisms including excitotoxicity, Ca^2+^ overload, caspases, and increased mitochondrial ROS [80,81]. Nevertheless, multiple clinical studies have reported conflicting reports as to whether the genotype of APOE genes influences the outcome of TBI [82,83,84,85,86,87]. There has been far less consideration for the genes encoded in mtDNA. One study has shown non-random association between APOE4 and mtDNA variants in sporadic Alzheimer’s disease [88]. Thus, there is some rationale for investigating the role of mtDNA variation and especially haplogroups in patients affected by TBI who carry a disease-associated APOE4 allele.

There are few reports, however, that have looked at mtDNA variation and TBI. One study looked at mtDNA variation in 880 subjects from Glasgow, Scotland who had survived TBI [89]. Using the Glasgow Outcome Scale (GOS) to quantify outcome six months after TBI, patients were categorized into four groups: good outcome, moderate disability, severe disability, and death or vegetative state. The study found that patients who had mitochondrial haplogroup K had significantly better outcomes than those who did not (odds ratio = 1.64, 95% confidence interval = 1.08–2.51, *p*-value = 0.02). There was no significant association when considering haplogroups H, J, T, and U. The study also suggested that mitochondrial haplogroups modulated the effect of age on TBI outcome. Aging is the primary risk factor for neurodegenerative disease [90], and age is negatively correlated with TBI outcome. But the study found that the strength of the relationship between TBI and age was different for patients having different mitochondrial haplogroups. The authors also found that patients who carried the APOE ε4 allele had significantly better outcome if they were also in haplogroup K compared to those who were not (odds ratio = 5.86, 95% confidence interval = 2.14–17.44, *p*-value = 0.002). These findings suggest an intricate interplay among mtDNA variation, pathophysiology of TBI, and aging.

Another study examined the correlation between mtDNA SNVs and TBI outcomes in 336 subjects up to one year, and found a significant association between TBI outcome and m.10398A > G in the *mt-ND3* mtDNA-encoded gene [91]. The A and G alleles at m.10398 were associated with slower and faster recovery, respectively. Furthermore, the A allele is associated with haplogroup H, while the G allele is associated with haplogroups K and J1. The study further reported that m.195T > C was associated with poorer TBI outcomes in female subjects, but not in male subjects. This result is consistent with the authors’ report that the T allele at m.195 was associated with mitochondrial dysfunction (*p* = 0.01) in female subjects only, and further supports a relationship between mtDNA variation and TBI outcome.

It is worth noting that the two aforementioned studies used a subset of mtDNA SNVs for interrogating mtDNA SNVs and did not employ NGS which would interrogate the entire mtDNA sequence. The use of NGS would likely improve the quality of the findings as NGS should identify a greater number of mtDNA variants with greater accuracy. Furthermore, studies investigating the impact of other forms of mtDNA variation, such as mtDNA-CN and mtRNA, are likely to provide additional insight into the role of mitochondrial function in TBI outcomes.

### 8.2. Ischaemic Stroke

Stroke can be broadly categorized into either ischaemic or haemorrhagic. Haemorrhagic stroke occurs in about 20% of stroke cases and is caused by blood vessel rupture. Ischaemic stroke results from an obstruction of blood supply in the brain, spinal cord or retina, and is responsible for approximately 80% of the remaining stroke episodes worldwide [92]. Ischaemic stroke entails a complex cascade of events, ultimately leading to cell damage and death. Typically, a blood clot or plaque forms in an artery supplying the brain, thereby causing a blockage of blood flow to the brain. Blood restriction causes a decrease in oxygen and glucose to tissues, leading to mitochondrial dysfunction [93,94]. Increased mitochondrial dysfunction results in decreased oxidative metabolism and ATP, coupled with an increase in toxic reactive oxygen and nitrogen species (ROS and RNS). This initial phase leads to neuronal death and inflammation and can only be stopped or reversed once blood flow resumes. Unfortunately, the return of blood to a tissue following ischaemia—reperfusion—also gives rise to tissue damage and is accompanied by an increase in ROS and RNS. Both ROS and RNS produce protein, lipid, and DNA damage, and can further damage mitochondria. If the mitochondria become highly damaged, proapoptotic molecules will be released by the mitochondria into the cytoplasm triggering additional cell death [93].

Given that ischaemic stroke invariably affects neuronal cells and the brain, it is not surprising that acute brain insults such as stroke and TBI increase the risk of a common neurodegenerative disorder, Alzheimer’s disease [95]. Many survivors of stroke and TBI develop neurodegenerative disease many years later [96]. There is also a clear connection between the pathology of ischaemic stroke and mitochondrial function. Therefore, it is natural to ask whether mtDNA plays a role in the development of pathogenesis and neurodegeneration due to ischaemic stroke.

Ostensibly, the most obvious connection between mtDNA variation and stroke is the primary mitochondrial disease: mitochondrial myopathy, encephalopathy, lactic acidosis, and stroke-like episodes (MELAS) [97]. The symptoms of MELAS, however, are diverse and broad, encompassing recurrent vomiting, migraine-like headaches, and stroke-like episodes [98]. Unlike ischaemic stroke, MELAS typically presents in children and young adults. MELAS is primarily associated with a single, heteroplasmic, point mutation in mtDNA as approximately 80% of patients with MELAS possess the m.3243A > G variant in the tRNA^Leu(UUR)^ gene. A change in a tRNA suggests that protein translation in the mitochondria is impaired, but the exact pathological mechanism is unknown [99].

There is, however, compelling evidence that mtDNA variation plays a role in susceptibility to ischaemic stroke. Several studies have implicated mitochondrial haplogroups in the risk for stroke. In one of the earliest large studies, mtDNA sub-haplogroup K was found to be at lower risk for transient ischaemic attack and isachaemic stroke compared to other haplogroups in a European population (odds-ratio 0.54, 95% confidence interval = [0.39–0.75]) [100]. In this study, however, only mtDNA variants were explored, and hence there was no accounting for population stratification. In another study, control-region (D-loop) variants m.16145 G > A and m.16311 T > C were found to be potential risk factors for stroke (*p*-value = 0.038 and *p*-value = 0.018, respectively) [101]. It should be noted that there is no mention of multiple-testing correction in this study, only the variants within the mitochondrial D-loop were considered, and there is no accounting for population stratification. One well-executed study enrolled 830 Taiwanese patients with a history of ischaemic stroke along with 966 healthy controls to assess the correlation between mtDNA haplogroup and risk for ischaemic stroke [102]. The study found that haplogroup F was at a significantly increased risk for ischaemic stroke (Bonferroni corrected *p*-value = 0.002, Odds Ratio = 1.44, 95% confidence interval = [1.14–1.82]), compared to haplogroups A, B, C, D, E, F, G, M7, M8, M9, other N, and other M. Further sub-haplogroup analysis revealed that haplogroup F1 was at a significantly higher risk of ischaemic stroke compared to sub-haplogroups B4, B4, D4, D5, F2, M7b, and M7c (Bonferroni adjusted *p*-value = 0.001, Odds Ratio = 1.72, 95% confidence interval l = [1.27–2.34]).

To reinforce these findings, the authors evaluated functional differences between haplogroup F1 and other haplogroups using cytoplasmic hybrids (cybrids) [103], which are a fundamental tool in mitochondrial functional experiments. Cybrids in the context of mitochondrial biology are cells that all have the same nuclear genome, but different mtDNA. In this way, the functional effect of differences among different mtDNA sequences can be evaluated using the same nDNA background. Cybrids are formed from eukaryotic cells which have been depleted of mtDNA, called rho-zero (ρ^0^) cells. These rho-zero cells are fused with cytoplasts (enucleated cells) to generate a cybrid. Cybrids are a valuable and powerful tool for assessing functional changes due to differences in mtDNA sequences. The authors created cybrids for all mitochondrial subgroups (B4, B4, D4, D5, F1, F2, M7b, and M7c) and found that while HIF-1α was elevated in all cybrids under hypoxic-ischemic stress, haplogroup F1 showed the lowest increase in HIF-1α. Furthermore, in a hypoxic environment, haplogroup F exhibited reduced protective responses and decreased mitochondrial function. Inflammatory cytokines, IL1, IL-6, and TNF-α were elevated in haplogroups F1 and F2. The implication of these findings is that haplogroup F is susceptible to the pathogenesis of ischaemic stroke compared to the other haplogroups investigated. To give further context, approximately 600 million people in the East Asia region have haplogroup F, so this finding potentially affects a large population.

A recent study assessed the correlation between mtDNA variation and cardiovascular disease using blood samples from 996 patients [104]. The authors reported that mtDNA-CN was associated with several cardiovascular disease phenotypes, including a history of myocardial infarction, and a history of coronary artery disease. Approximately 5% of these patients had a history of stroke, but no significant correlation was found between mtDNA-CN and stroke. Although these results are intriguing and point to a pivotal role of mtDNA in cardiovascular disease pathology, there are some technical caveats. First, there was no report of accounting for NUMTs in this analysis, which may confound the copy number calculation. Second, the differences in mtDNA-CN in this study represent the collective differences in the ratios of all distinct types of white blood cells. Hence, differences in observed mtDNA-CN may in fact be due to changes in the ratios of the different white blood cell types. To determine the relative composition of granulocytes, monocytes, and leukocytes would require approaches comparable to cell sorting, which are not trivial to implement. Hence, care must be taken in interpreting such results as differences in mtDNA-CN from blood samples could actually reflect potential changes in the composition of white blood cell types.

So far, we have discussed inherited mtDNA variation as a risk factor in neurodegenerative disease. Once a neuronal injury occurs, mtDNA variants and decreased mtDNA-CN can arise as a consequence of the injury, however. Thus, healing and repair of these neurons including repairing and restoring mtDNA may also play a vital role in determining the outcome of a pathology. Damaged mtDNA is common in various neurodegenerative diseases including Alzheimer’s disease and Parkinson’s disease [105,106]. In atherosclerosis—a common cause of ischaemic stroke–mtDNA damage in leukocytes was associated with higher-risk plaques [107]. Increased mtDNA damage may also lead to a decrease in mtDNA-CN; a meta-analysis found that mtDNA-CN is lower in individuals with cardiovascular disease compared to controls, and that a lower blood mtDNA-CN increases the likelihood of cardiovascular events [108]. MtDNA damage can occur as a result of ROS, UV-light, and chemical compounds [109], and lesions that are left uncorrected can become cytotoxic [110]. There are several repair pathways associated with mtDNA, and the best understood and primary repair mechanism is base excision repair (BER), [109,111]. Typically, a lesion occurs at a region in the mtDNA leaving a site that is neither a purine nor a pyrimidine, referred to as apurinic and apyrimidinic, respectively. BER involves identifying, excision and replacing a damaged base at an abasic (either apurnic or apyrimidinic) site (Reviewed in [109,111,112,113]). While the BER mechanism for repair acts on both nDNA and mtDNA, all BER-related enzymes are encoded in the nDNA.

Less efficient BER in mtDNA increases the likelihood of cytotoxicity [114], and several human disease syndromes, including neurodegeneration, have been associated with defects in BER pathways [109]. Dysfunction in BER pathways has also been reported in the brains of patients with Alzheimer’s disease [115]. In animal models of ischaemic stroke, proteins associated with BER are up-regulated [109], and administering mitochondrial BER enzyme attenuates infarct size and neurologic injury, reducing reperfusion injury [116]. These findings support a role for BER in promoting recovery after neuropathologies; however, some studies have suggested that BER may actually contribute to brain damage in ischaemia [109], and Parkinson’s disease [117]. Thus, BER likely contributes to both recovery and pathology in neurogenerative disease.

## 9. Future Directions and Conclusions

There is accumulating evidence demonstrating that mitochondria underlie the pathology of common, non-primary mitochondrial disease. In secondary neurodegenerative disease, the mitochondrion is one component of a complex and multifactorial cascade of events. There is ample evidence to suggest that properly functioning mitochondria are necessary for recovery following secondary neurodegenerative disease. Understanding the complex and diverse aspects of mitochondrial genetics and genomics will aid in further research into understanding and treating secondary neurodegenerative disease. Third generation sequencing, such as HiFi reads produced by the PacBio Sequel IIe [118], offers an opportunity to resolve some of the concerns raised here, in particular the issue of NUMTs. The long reads produced by HiFi sequencing, averaging 20 kb or more will ameliorate some issues due to inaccurate sequence alignment due to NUMTs. A remarkable amount of progress has been made in diagnosing mitochondria-related disease. With further understanding of disease etiology and disease-associated variants in the context of mitochondria, there is promise for improving care for secondary neurodegenerative disease.
